# Hepatitis Free Hospital: Retrospective Results of an In-Hospital Project of Hepatitis C Virus Micro-Elimination

**DOI:** 10.3390/v18050516

**Published:** 2026-04-30

**Authors:** Federica Cerini, Loreta A. Kondili, Chiara Masellis, Agostino Cosenza, Carmelo Selvaggio, Maria Grazia Rumi

**Affiliations:** 1 Hepatology Unit, San Giuseppe Hospital, University of Milan, 20123 Milan, Italy; chiara.masellis@unimi.it (C.M.); agostino.cosenza@unimi.it (A.C.); carmelo.selvaggio@unimi.it (C.S.); mariagrazia.rumi@unimi.it (M.G.R.); 2 Department of Clinical Sciences and Community Health, Dipartimento di Eccellenza 2023–2027, University of Milan, 20122 Milan, Italy; 3 Centre for Global Health, Istituto Superiore di Sanità, 00161 Rome, Italy; loreta.kondili@iss.it; 4 UniCamillus-Saint Camillus International University of Health Sciences, 00131 Rome, Italy

**Keywords:** hepatitis C virus, in-hospital screening, opportunistic screening, micro-elimination, linkage to care, case finding

## Abstract

National HCV screening, a cost-effective program in Italy, is supported by the Italian Ministry of Health. Free-of-charge screening was approved for people who use drugs, prisoners and the 1969–1989 general population birth cohort. However, the benefits of extending hospital-based screening irrespective of age, in terms of case detection and linkage to care, remain unclear. We assessed a streamlined in-hospital pathway at San Giuseppe Hospital (Milan), between 2021 and 2024, with anti-HCV screening for all admissions and HCV-RNA confirmation when indicated. Anti-HCV seroprevalence among 18,218 screened patients was 1.9% (medical wards 3.8%, surgical 1.4%). Viremia was detected in 23/75 tested (31%); 78% linked to care and 30% initiated treatment. Notably, 91% of viremic patients were born before 1969 indicating hospital-based screening effectively identifies HCV infections in older cohorts missed by national screening programs. Systematic screening implementation across hospital wards represents a pragmatic micro-elimination strategy for Italy and similar epidemiological contexts, advancing 2030 elimination targets.

## 1. Introduction

In 2015, the World Health Organization (WHO) launched a global plan to eliminate hepatitis C as a public health threat, setting targets to reduce HCV-related mortality by 65% and new infections by 90%. Achieving these goals relies on the availability of highly effective, well-tolerated direct-acting antivirals (DAAs), which achieve cure rates exceeding 95% [1,2]. Nonetheless, nearly a decade after the widespread commercialization of these therapies, a substantial proportion of viraemic patients remains in Europe, with the potential to cause significant future health and socioeconomic consequences [3].

Italy has historically reported the highest prevalence of HCV infection and liver disease–related mortality in Europe, driven by a marked cohort effect due to early nosocomial transmission and a later epidemic wave associated with intravenous drug use [4]. Although mass screening represents a cost-effective and ideal strategy for achieving elimination, sustainability and feasibility constraints have required a graduated approach [5]. Accordingly, in 2021, Italy implemented a publicly funded nationwide screening program targeting key populations and individuals born between 1969 and 1989, prioritized to reduce ongoing transmission for more at risk for key populations that mostly belong to the 1969–1989 cohort [6]. While this represents an important initial measure, extension of screening to the broader 1948–1988 birth cohort is required in Italy to meet WHO targets, as supported by Italian serosurveys and modeling studies [6,7,8]. Beyond screening expansion, strengthening linkage to care is essential to translate case detection into meaningful public health impact, as loss to follow-up occurs at multiple steps of the HCV care cascade and remains a major barrier to elimination [9]. Micro-elimination offers a complementary approach by targeting populations in which prevention and treatment can be implemented more effectively [10,11]. In this context, hospital-based screening programs may represent a valuable strategy, although evidence on their effectiveness and feasibility remains limited to only a few pilot projects in different hospitals in different Italian regions.

This brief report describes the implementation of a hospital-based HCV screening program at San Giuseppe Hospital (Milan, Lombardy), between 2021 and 2024, aimed at assessing the feasibility and the impact of a streamlined referral pathway on the identification and treatment of HCV infection and its potential contribution to HCV elimination efforts.

## 2. Methods

From February 2021 to March 2024, a hospital-based universal screening for anti-HCV antibodies was carried out in San Giuseppe Hospital named “HCV-free Hospital project”. According to this project, all patients (regardless of age and reason for hospitalization) consecutively admitted to the medical or surgical wards or Day Hospital/Day Surgery were tested for anti-HCV antibodies. In the case of HCV Ab-positivity, tests for HCV-RNA to confirm chronic infection have been performed. Patients admitted to COVID-19 wards were not included in the screening program while those admitted to the Hepatology Unit were excluded, as they routinely underwent disease-specific HCV screening. For individuals with multiple hospital admissions, only the first admission was included in the analysis.

During the project, the Information Technology department generated a report twice a week, tracking all patients who underwent anti-HCV testing. This report included patient data, hospital ward, the test date and results, and, where applicable, the results of HCV-RNA tests.

Patients diagnosed with chronic hepatitis C were referred to the Hepatology Unit for clinical assessment and evaluation for antiviral treatment with DAAs. Clinical evaluation in eligible patients included evaluation of liver stiffness measurement using transient elastography. Data on potential routes of HCV transmission, including intravenous drug use, blood transfusions prior to the 1990s, and use of non-disposable glass syringes, were collected by physicians as part of the routine anamnestic interview performed at the time of clinical assessment.

Categorical variables were reported as frequencies (percentages) and continuous variables (age) as mean standard deviation [SD]). Categorical variables were compared using the Chi-square tests to assess differences in anti-HCV detection rates between males and females, across birth cohorts, and between ward types.

The study (number 4183/2025) was approved by the Local Ethics Committee of the Hospital (CET Lombardia 5), which waived the requirement for informed consent due to the retrospective and anonymized nature of the data.

## 3. Results

Over a 3-year period, 18,218 hospitalized patients were screened for HCV-Ab (mean age 53.6 years ± 21.7 and 58% female) (Table 1). Of these, 355 were HCV-Ab positive, corresponding to an overall prevalence of 1.9%, with a higher rate among males (2.5% versus 1.5%, *p* < 0.001). Age-stratified analyses showed the highest anti-HCV prevalence in older birth cohorts, reaching 3.9% among individuals born before 1948 and 2.7% in those born between 1948 and 1968, and declining in the 1969–1989 (0.8%) and post-1989 (0.4%) cohorts (*p* < 0.001). Anti-HCV positivity was higher among subjects admitted to medical wards (3.8%) than among those admitted to surgical wards (1.4%) (*p* < 0.001) (Table 1).

Among 355 anti-HCV positive patients, 45.6% (162/355) were ineligible for further evaluation: previous SVR (n = 104, 64.2%), high comorbidity/low life expectancy (n = 41, 25.3%), deceased (n = 12, 7.4%), false positive (n = 3, 1.9%), and discharge against medical advice (n = 2, 1.2%). HCV-RNA testing was not performed in a further 33.2% (118/355) of patients because they were discharged from the hospital prior to the HCV-RNA determination.

**Figure 1:**
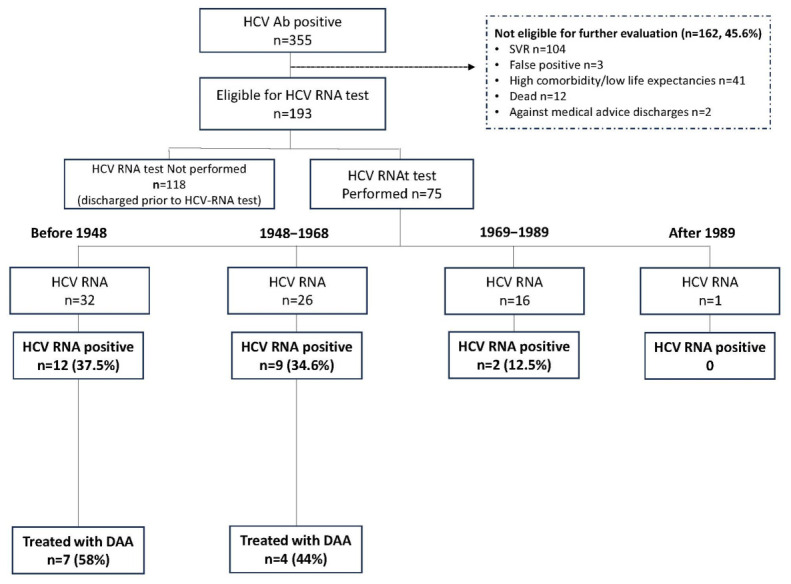
Flowchart of the in-hospital HCV screening program.

Of the 75 individuals who underwent HCV RNA testing, active HCV infection was identified in 30.7% (23/75), corresponding to an overall prevalence of 0.12%, and 48 had undetectable viremia (in 4 patients a HCV-RNA test was performed in the region of origin, so these data were not available). Linkage to care was achieved in 18 cases (18/23, 78.3%), and 11 (11/18, 61.1%) had already completed treatment with DAAs reaching SVR (Figure 1). At baseline, patients included in the study had a moderate hepatic fibrosis, with a mean liver stiffness value of 7.06 kPa (range: 3.5–13.2 kPa). According to birth cohort, most HCV-RNA positive cases occurred among individuals born before 1948 (12/32, 37.5%) and between 1948 and 1968 (9/26, 34.6%), with a higher proportion of unknown HCV RNA status before hospital admission in these older cohorts (75.0% and 66.7%, respectively). Regarding the identified routes of infection, intravenous drug use, blood transfusions prior to the 1990s, and the use of non-disposable glass syringes for pharmacological treatments were reported.

## 4. Discussion

In the context of HCV elimination efforts, our findings provide additional evidence on the burden of chronic HCV infection in an unselected hospitalized population and support the feasibility of hospital-based screening for effective linkage to care and treatment initiation. In a country such as Italy, where the HCV burden is declining but an estimated 100,000 individuals remain in need of treatment identification, hospital-based approaches may play an important role in comprehensive elimination strategies [8,12,13].

In our study, anti-HCV positivity was identified in 1.9% of screened patients. Among anti-HCV positive individuals with available HCV RNA testing, approximately 31% were viraemic patients. A proportion of anti-HCV–positive patients (64%) had an undetectable viral load, likely due to spontaneous viral clearance and in a few cases due to unreported antiviral treatment. These findings are consistent with previously published opportunistic screening studies, which reported rates of undetectable HCV RNA among anti-HCV-positive individuals ranging from 64% to 82% [14,15]. Notably, a substantial proportion of anti-HCV–positive patients (33.2%, 118/355) did not undergo HCV RNA testing. These results align with previously reported gaps in the HCV care cascade in Italian hospital settings, where a significant proportion of anti-HCV-positive patients remains unlinked to confirmatory virological testing, largely due to barriers such as premature discharge, patient refusal, or failure to recall [16,17]. Addressing this gap is essential to accurately identify viraemic individuals who could benefit from antiviral treatment, in line with the national HCV elimination strategy [10]. The HCV reflex test introduction may prevent the need for a two-step diagnostic process with a confirmatory test automatically performed if the first test result is positive. Anti-HCV prevalence showed a clear age-related increase, rising from 0.4% in individuals born after 1989 and 0.8% in those born between 1969 and 1989 to 2.7% in the 1948–1968 cohort and 3.9% among those born before 1948, confirming Italy’s cohort effect [7,18]. Among the 23 HCV RNA–positive patients, 91% (21/23) were born before 1969, outside the 1969–1989 birth cohort currently targeted by national free HCV screening programs, in line with recent findings reported by Ferrarese et al. [14]. Taken together, these data underscore the need to extend screening and case finding beyond the currently targeted 1969–1989 birth cohort to enhance identification of active infections to be treated. Of note, we also found that approximately 70% of viraemic patients were previously unaware of their infection, highlighting a significant reservoir of undiagnosed HCV among individuals born before 1969. Conversely, 30% had prior HCV diagnosis but remained untreated despite effective therapies being available. This dual pattern, undiagnosed infection and treatment gaps, underscores that both enhanced case identification and strengthened linkage to care are essential for achieving 2030 elimination targets.

Therefore, the identification of patients with active HCV infection in hospital settings may facilitate more rapid and effective linkage to care and treatment, compared with the ongoing HCV screening of general population that has reported lower linkage-to-care rates [5]. In our experience, 78% (18/23) of viremic patients were successfully linked to specialist services, supporting hospital-based approaches as viable strategies for effective HCV linkage to care [19]. Furthermore, we found that treatment was completed in 61% (11/18) of viremic patients. Among those who did not receive treatment, reasons included preference for continuation of care at local healthcare facilities, severe comorbidities, in-hospital death, and patient refusal. The latter often reflected insufficient awareness of HCV severity and treatment benefits, highlighting the need for patient education initiatives that emphasize disease progression risks and the high efficacy of current curative therapies [9].

Interestingly, HCV seroprevalence was 2.7-fold higher in medical versus surgical wards (3.8% vs. 1.4%), reflecting the concentration of older, high-risk patients in medical settings. These findings highlight the importance of increasing awareness among healthcare professionals and strengthening infection control measures within hospital settings [12,14]. Recent studies have documented a substantial contribution of intra-hospital HCV transmission, including patient-to-patient transmission [20]. In this context, and given the higher prevalence of HCV infection among older individuals who are more frequently hospitalized, systematic in-hospital screening could contribute to earlier identification of undiagnosed infections and to reducing the risk of onward transmission within healthcare settings.

It should be noted that one limitation of the study is that detailed patient data on individual HCV risk factors were not collected; however, the observed age distribution of infections is consistent with established epidemiological patterns in Italy [7,14]. Approximately one third of anti-HCV-positive patients did not undergo confirmatory HCV RNA testing, reflecting losses inherent to the two-step diagnostic process. Future programs should consider the involvement of a dedicated staff to improve patient monitoring and prevent loss to follow-up across the care cascade. In addition, the adoption of HCV reflex testing could substantially reduce drop-out between diagnostic steps.

In conclusion, in addition to the ongoing screening the 1969–1989 birth cohort, the progressive decline in the prevalence of active HCV infection in Italy, and the low coverage achieved by screening initiatives in the general population, these data strongly indicate that hospital-based screening may represent a strategic and pragmatic approach to address the residual burden of active HCV infection, particularly among older individuals who are currently not adequately reached by targeted case-finding strategies. Yet, gaps in confirmatory testing among eligible patients indicate that strengthening the referral pathway remains a priority to maximize the public health impact of hospital-based screening. These findings may be relevant not only for Italy, but also for other countries with similar HCV epidemiological profiles, supporting progress toward HCV elimination by 2030 through strategies that are both feasible and sustainable. 

## Figures and Tables

**Table 1:** Characteristics of screened population and HCV-Ab positive patients.

	Screened Patients	HCV-Ab Positive	
	N (%)	n (% ^1^)	% ^2^
Overall	18,218	355	1.9
Female	10,566 (58.0)	162 (45.6)	1.5
Male	7652 (42.0)	193 (54.4)	2.5
Mean age ± SD	53.6 ± 21.7	69 ± 16.3	
Year of birth			
<1948	3926 (21.6)	154 (43.3)	3.9
1948–1968	5391 (29.6)	146 (41.1)	2.7
1969–1989	5552 (30.5)	43 (12.1)	0.8
>1989	3349 (18.4)	12 (3.4)	0.4
Medical wards	3829 (21.0)	147 (41.4)	3.8
Surgical wards	14,389 (79.0)	208 (58.6)	1.4

^1^ Out of the HCV-Ab positive patients. ^2^ Out of the screened population.

## Data Availability

Data, analytic methods and study materials will be made available to other researchers upon reasonable request.

## Abbreviations

The following abbreviations are used in this manuscript:

WHO	World Health Organization
HCV	Hepatitis C Virus
DDA	Direct-acting antiviral
